# Childhood Trauma, Cognitive Emotion Regulation and Motivation for Behavior Change Among Clients of Opioid Substitution Treatment With and Without Past Year Synthetic Cathinone Use During Therapy

**DOI:** 10.3389/fnins.2020.00037

**Published:** 2020-01-31

**Authors:** Máté Kapitány-Fövény, Anna Kiss, Judit Farkas, Kinga Edit Kuczora, Patrícia Pataki, Janka Horváth, Zsolt Demetrovics

**Affiliations:** ^1^Faculty of Health Sciences, Semmelweis University, Budapest, Hungary; ^2^Nyírõ Gyula National Institute of Psychiatry and Addictions, Budapest, Hungary; ^3^Institute of Psychology, Eötvös Loránd University, Budapest, Hungary

**Keywords:** NPS, synthetic cathinone, opioid substitution therapy, trauma, emotion regulation

## Abstract

**Background:**

With a decrease in heroin’s purity and availability in the European drug market, Hungarian opioid dependent patients started to substitute heroin with novel psychoactive substances (NPS) and especially with synthetic cathinones.

**Goal:**

This study aims to assess whether clients of opioid substitution treatment (OST) with and without a history of synthetic cathinone use during therapy differ in (1) the rate and type of experienced childhood trauma, (2) the way they cope with negative life events, (3) their motivation to change substance use behavior, (4) the rate of treatment retention.

**Methods:**

A total of 198 clients of an outpatient centers (Nyírõ Gyula National Institute of Psychiatry and Addictions, Budapest) OST were asked to provide information about their general substance use experiences, including the consumption of NPS during treatment, their childhood traumatic experiences (Childhood Trauma Questionnaire), cognitive emotion regulation strategies (Cognitive Emotion Regulation Questionnaire), their motivation to change substance use behavior (University of Rhode Island Change Assessment Scale) and current psychiatric symptoms (Brief Symptom Inventory). Baseline data was collected in the summer of 2015, while 4 years follow-up data on treatment retention was obtained in the summer of 2019.

**Results:**

The majority of the clients were male (*N* = 141, 71.2%), receiving methadone as a substitute for opioids (*N* = 178, 89.9%), while mean age of the full sample was 39.7 (SD = 6.8). Based on a logistic regression model, the odds for past year synthetic cathinone use was higher among clients with more severe psychiatric symptoms (*B* = 0.8, OR = 2.2, *p* < 0.01) and among clients who were in treatment for a shorter period of time (*B* = 0.1, OR = 0.9, *p* < 0.05). Synthetic cathinone use during treatment was further associated with less adaptive strategies to cope with negative life events. Synthetic cathinone use was also a risk factor for reduced treatment retention (*B* = −0.8, OR = 0.4, *p* < 0.05) and was associated with lower odds of being member of a latent class with less severe psychopathological profile (*B* = −0.9, OR = 0.4, *p* < 0.05).

**Conclusion:**

Synthetic cathinone use during treatment is associated with poorer treatment outcomes and might be characterized by more severe psychiatric symptoms and amotivation to change substance use among opioid dependent clients.

## Introduction

Considering the ever-changing world of contemporary drug scene, the past two decades were characterized by the emergence of NPS, started with the appearance of SC, as first reported in the early 2000s (Urquhart, 2004). Cathinone-derivatives, such as mephedrone, 4-MEC, methylone, pentedrone or MDPV became popular alternates of formerly scheduled psychostimulants and even depressants (e.g., opioids). Regular SC users usually attributed their consumptive choices to easy availability, perception of safety, low prices and pleasurable drug effects (e.g., Van Hout and Brennan, 2012). Although most countries have implemented some forms of control legislations, varying in their type or depth (Corazza and Roman-Urrestarazu, 2018), online trafficking that provides relative anonymity for NPS users (EMCDDA, 2016) and the fact that NPS consumption is now an integral part of the drug scene still support SC popularity among members of certain subpopulations (such as club-goers, prisoners, psychiatric patients, and injecting drug users). Based on the results of the European Syringe Collection and Analysis Project Enterprise (ESCAPE) 2017 campaign (EMCDDA, 2019), traces of SC were found in a high proportion of the 1521 syringes collected from six sentinel European cities (Amsterdam, Budapest, Glasgow, Helsinki, Lausanne, and Paris), indicating that SC use among people who inject drugs is a continuous phenomenon.

Injecting SC use was reported as a low-level and localized incident in many European countries, except for Hungary and Romania where a more substantial level of SC injecting was observed. Injection of SC is of decisive relevance among those who are injectors of other psychoactive substances, including opioids and amphetamines (EMCDDA, 2015). Certain reports denoted that long-term abstinent ex-opiate users shifted to SC injecting (e.g., Van Hout and Bingham, 2012; Péterfi et al., 2014; Rácz et al., 2015) throughout a time period of sustained heroin shortage in the drug market. As part of this shift, clients of OST started to abuse SC during their maintenance treatment (Heikman et al., 2017; Kapitány-Fövény et al., 2017), explained by either treatment deficiencies (inadequate dose of OST medicine), easy SC availability or specific individual factors (e.g., severity of psychiatric symptoms).

From a clinical perspective it is of high relevance to understand the potential reasons of substance use during treatment among OST clients, and more specifically to explore whether or not these reasons differ by the substance being consumed. The local phenomenon of opioid dependent patients’ SC use enables the study of SC-specific consumption determinants in a clinical sample. Of the many possible factors underlying substance use during OST – e.g., shorter treatment duration (Li et al., 2012), a medium 60–100 mg/day medication dose (Baumeister et al., 2014), low treatment attendance, drug using friends, family conflicts (Sullivan et al., 2014), male gender (Vigna-Taglianti et al., 2016), younger age (Amiri et al., 2018), or the quality and supportiveness of the social network (Shen et al., 2018) – this paper focuses on the presumed impact of unresolved childhood trauma, with special emphasis placed on individual patterns of cognitive emotion regulation as related to stressful live events.

Posttraumatic stress disorder frequently co-occurs with SUD. An estimated prevalence of PTSD in SUD patients may range from 25 to 49%, a three times higher prevalence than that in the general population (Gielen et al., 2012). PTSD and SUD may result in shared pathological features, such as heightened stress sensitivity, reward deficiency or impulsive behavior with overlapping impaired neurocircuitry (Enman et al., 2014). Furthermore, as both pathologies can be interpreted as the outcomes of hyperactivity to reminding cues, some argues that PTSD and SUD are in fact disorders of memory (Gisquet-Verrier and Le Dorze, 2019). Exposure to either stress or psychoactive substances can induce epigenetic alterations resulting in similar neurobiological, molecular and behavioral changes (e.g., glucocorticoids can induce excitation patterns by activating dopamine neurons) that facilitate drug-seeking behavior (Pizzimenti and Lattal, 2015). However, experiencing traumatic events does not necessarily lead to PTSD. Emotion regulation – connected to cognitive processes – helps the individual in recognizing, evaluating and influencing the course and expression of emotions (Domaradzka and Fajkowska, 2018). Posttraumatic cognitive emotion regulation plays an important mediatory role in the relationship between trauma and either PTSD or PTG. For instance, intrusive rumination might be associated with PTSD, while deliberate rumination can be linked to PTG (Zhang et al., 2018), indicating different cognitive processes in case of negative or positive trauma outcomes, even if PTSD and PTG may also coexist on an individual level. Similarly, while some forms of cognitive processes are thought to be unproductive (e.g., overgeneralization, avoidance), there are many constructive ways (e.g., decentering, accommodation of corrective information) of trauma processing (Hayes et al., 2017).

Regarding the subpopulation of OST clients, early childhood trauma has been linked to poorer treatment retention in OST programs (Kumar et al., 2016), childhood sexual abuse was found to be a predictor of drug use during OST (Schiff et al., 2010), while NPS use among treatment seeking opioid dependent patients was evaluated as an attempt of self-medication for earlier psychological trauma (Gittins et al., 2018). Additionally, OST patients with a history of childhood trauma show impaired ability to associate a stimulus with its outcome when the stimuli is presented in a drug-related context (Weiss et al., 2019), hence clients’ cognitive efficacy might be reduced by drug-related stimuli. Cues that are associated with either stress or drugs also increase opioid craving and anxiety (Hyman et al., 2007) and pose negative impact on OST prognosis (Jaremko et al., 2015).

As regards SC use among OST clients, the subgroup of patients who consume any SC during treatment is characterized by younger age, shorter treatment duration, more severe psychiatric symptoms and higher stress reactivity to interpersonal conflicts (Kapitány-Fövény et al., 2017). Based on these previous findings, the current study aimed to assess whether OST clients with and without a history of SC use during treatment differ in experienced childhood trauma, cognitive emotion regulation strategies (i.e., how they cope with stressful life events), the severity of their psychiatric symptoms, their motivation for behavioral change and the rate of treatment retention.

## Materials and Methods

### Procedure

A total of 206 clients of Hungary’s biggest drug outpatient centers (Nyírõ Gyula National Institute of Psychiatry and Addictions, Budapest) OST were involved in the study. Data collection was implemented by face-to-face interviews conducted by trained psychologists. Inclusion criteria were: (1) being a client of either methadone or Suboxone (a combination formulation of buprenorphine and naloxone) maintenance therapy for at least 1 year, (2) 18 < years of age, (3) signing the informed consent form; while exclusion criteria were: (1) acute drug effects, (2) agitation or violent behavior. After eight patients were excluded from the study, the final sample consisted of 198 participants. In order to prevent respondents from being influenced by the acute sedative effects of OST medication, data was collected just before the administration of either methadone or buprenorphine-naloxone.

Baseline data was collected in the summer of 2015, while follow-up data on treatment retention was obtained in the summer of 2019 (4 years follow up).

Ethical approval was provided by the hospital’s Research Ethics Committee.

### Measures

#### Demographics and Treatment Characteristics

Demographic items were comprised of questions regarding gender, age, educational background, marital status, perceived SES and occupation. Perceived SES was measured by a 7-point Likert scale (from 1 = the lowest possible SES to 7 = the highest possible SES). Treatment indices included the length of treatment, the type of OST medication (methadone vs. buprenorphine-naloxone) and medication dose. The following rule-breaking behaviors were also explored: buying street methadone/buprenorphine-naloxone, intravenous administration of methadone/buprenorphine-naloxone.

#### Synthetic Cathinone Use

Participants provided information about their past year and past month SC use experiences during OST. Mephedrone, pentedrone and MDPV as the most prevalent SC in Hungary were listed in the questionnaire.

#### Childhood Trauma

Childhood traumatic experiences (CTQ) were assessed by the short form of the CTQ-SF. CTQ-SF is a retrospective recall based measure (Bernstein et al., 2003), containing 28 items of which three are validity items. The remaining 25 clinical items load on five factors: (1) Emotional abuse (e.g., “*Family said hurtful things*”), (2) Physical abuse (e.g., “*Punished with hard objects*”), (3) Sexual abuse (e.g., “*Was sexually abused*”), (4) Emotional neglect (e.g., the reversed item of “*Made to feel important*,” (5) Physical neglect (e.g., “*Not enough to eat*”). Respondents evaluate each statement by using a 5-point Likert scale (from 0 = never to 4 = very often).

Good reliability was found regarding the five factors, with the following Cronbach’s α scores: Emotional abuse = 0.89, Physical abuse = 0.92, Sexual abuse = 0.94, Emotional neglect = 0.95, Physical neglect = 0.87.

#### Cognitive Emotion Regulation

The short 18-item version of the CERQ (Garnefski and Kraaij, 2006; Miklósi et al., 2011) was applied to measure subjects’ stress-related cognitive-affective processing style. The short form of the CERQ comprises of nine conceptual scales: (1) Self-blame (e.g., “*I feel that I am the one to blame for it*”), (2) Other-blame (e.g., “*I feel that others are responsible for what has happened*”), (3) Rumination (e.g., “*I dwell upon the feelings the situation has evoked in me*”), (4) Catastrophizing (e.g., “*I continually think how horrible the situation has been*”), (5) Positive refocusing (e.g., “*I think about pleasant experiences*”), (6) Refocus on planning (e.g., “*I think about how to change the situation*”), (7) Positive reappraisal (e.g., “*I think I can learn something from the situation*”), (8) Putting into perspective (e.g., “*I think that it all could have been much worse*”) and (9) Acceptance (e.g., “*I think that I have to accept the situation*”), with two items per scale. The questionnaire’s items are evaluated on a 5-point Likert scale (from 1 = almost never to 5 = almost always). The higher the subscale score, the more inherent the cognitive strategy is. CERQ’s conceptual scales are additionally interpreted as either adaptive (Positive refocusing, Planning, Positive reappraisal, Putting into perspective and Acceptance) or non-adaptive (Self-blame, Other-blame, Rumination, Catastrophizing) strategies.

With regard to reliability testing, the following good, acceptable or low Cronbach’s α scores were found for the nine scales: Self-blame = 0.63, Other-blame = 0.72, Rumination = 0.83, Catastrophizing = 0.82, Positive refocusing = 0.68, Refocus on planning = 0.63, Positive reappraisal = 0.62, Putting into perspective = 0.53, Acceptance = 0.67.

#### Motivation for Behavior Change

Respondents’ current motivational state regarding a potential decision to change their substance use habits (e.g., stop using any psychoactive substances during treatment or reducing their OST medication dose) was measured by the 32-item version of the URICA (DiClemente and Hughes, 1990). URICA is based on the TTM of behavioral change (Prochaska and DiClemente, 1983) and as such theoretically driven measure, contains the following subscales: (1) Precontemplation (e.g., “*I’m not the problem one. It doesn’t make much sense for me to consider changing*”), (2) Contemplation (e.g., “*I’ve been thinking that I might want to change something about myself*”), (3) Action (e.g., “*I am really working hard to change*”) and (4) Maintenance (e.g., “*I’m struggling to prevent myself from having a relapse of my problem*”). Readiness to change as a second-order factor is yielded by using the formula: Contemplation + Action + Maintenance - Precontemplation.

Responses are given on a 5-point Likert scale (from 1 = strongly disagree to 5 = strongly agree).

In the current study, the following low or good Cronbach’s α scores were observed: Precontemplation = 0.53, Contemplation = 0.49, Action = 0.79, Maintenance = 0.73.

#### Psychiatric Symptoms

Current (last week’s) psychiatric symptoms were assessed by the BSI (Derogatis, 1975; Urbán et al., 2014). The 53 items of the BSI are evaluated by using a 5-point Likert scale (from 0 = not at all to 4 = extremely). BSI comprises nine symptom scales: (1) Somatization (e.g., “*Pain on heart or chess*”), (2) Obsession-compulsion (e.g., “*Feeling blocked in getting things done*”), (3) Interpersonal sensitivity (e.g., “*Your feeling being easily hurt*”), (4) Depression (e.g., “*Feeling lonely*”), (5) Anxiety (e.g., “*Feeling tense or keyed up*”), (6) Hostility (e.g., “*Getting into frequent arguments*”), (7) Phobic anxiety (e.g., “*Feeling afraid to travel on buses, subways or trains*”), (8) Paranoid ideation (e.g., “*Feeling that you are watched or talked about by others*”) and Psychoticism (e.g., “*The idea that something is wrong with your mind*”). A GSI was computed as the mean of the 53 items.

Good or acceptable reliability results were found: Cronbach’s α scores regarding the nine scales were: Somatization = 0.87, Obsession-compulsion = 0.8, Interpersonal sensitivity = 0.73, Depression = 0.86, Anxiety = 0.85, Hostility = 0.82, Phobic anxiety = 0.78, Paranoid ideation = 0.62, and Psychoticism = 0.66.

#### Urine Samples

As part of the OST protocol, clients were randomly screened for the following substances by applying standard rapid urine tests: opiate, cocaine, amphetamine, MDMA, THC, benzodiazepine. Randomization was done by the hospital’s medical software as based on clients’ Treatment Demand Indicator (TDI) codes. Past year’s positive or negative test results were entered in our database retrospectively. Since the outpatient centers urine tests could not be used to detect SC, past year SC use was based on respondents’ report only (see section “Synthetic Cathinone Use”). Questionnaire data and urine test results were matched by clients’ TDI codes, however, in the final database only unique identifiers were recorded in order to protect personality rights.

### Statistical Analysis

Data were analyzed by SPSS 17 (SPSS Inc, 2008). Demographic characteristics and treatment indices were explored by descriptive statistics, potential differences between SC and non-SC-using clients were analyzed by non-parametric Mann Whitney *U* test and chi-square statistics, the association between childhood trauma types and non-adaptive cognitive emotion regulation strategies was tested by linear regression model, while significant predictors of past year SC use and treatment retention were identified by logistic regression models. Participants were classified by their highest standardized *z* score achieved on any of the BSI factors, referred to as “*prominent psychopathological dimension*,” based on a very similar approach presented by Maremmani et al. (2016). Psychopathological symptom profiles were explored by using latent class analysis in Mplus v5 (Muthén and Muthén, 1998–2007). Finally, predictors of latent class memberships were examined by logistic regression models.

## Results

### Sample Characteristics

Table 1 summarizes detailed sample characteristics in terms of demographics and treatment indices.

**TABLE 1:** Demographics and treatment characteristics.

**Demographics**		
Age Mean (SD)		39.7 (6.8)
Gender distribution	Male *N* (%)	141 (71.2)
	Female *N* (%)	57 (28.8)
Educational background	Elementary *N* (%)	52 (26.3)
	Vocational/technical school *N* (%)	91 (46)
	High school *N* (%)	28 (14.1)
	Incomplete higher education *N* (%)	8 (4)
	Completed higher education *N* (%)	19 (9.6)
Marital status	Single *N* (%)	120 (60.6)
	In a relationship *N* (%)	33 (16.7)
	Married *N* (%)	24 (12.1)
	Divorced *N* (%)	19 (9.6)
	Widowed *N* (%)	2 (1)
Perceived socioeconomic status Mean (SD)		3.9 (1.1)
Occupation	Unemployed *N* (%)	42 (21.2)
	Temporary job *N* (%)	23 (11.6)
	Permanent job *N* (%)	116 (58.6)
	Maternity leave *N* (%)	2 (1)
	Disability pension *N* (%)	14 (7.1)
	Student *N* (%)	1 (0.5)
**Treatment indices**		
Length of treatment Mean (SD)		6.9 (5.7)
Type of OST medication *N* (%)	Methadone *N* (%)	178 (89.9)
	Buprenorphine-naloxone *N* (%)	20 (10.1)
OST medication dose (mg)	Methadone Mean (SD)	77.9 (33.8)
	Buprenorphine-naloxone Mean (SD)	8.6 (5.5)
Buying street methadone/buprenorphine-naloxone *N* (%)		98 (49.5)
Intravenous administration of methadone/		99 (50)
buprenorphine-naloxone *N* (%)		
4 year treatment retention rate *N* (%)		74 (37.4)

Clients with and without a history of past year SC use were compared regarding demographic and treatment-related variables. SC using clients were younger [SC-using: M = 37.1, SD = 6.6, non-SC-using: M = 40.5, SD = 6.6, *t*(196) = 2.9, *p* < 0.01], being in treatment for a shorter period of time [SC-using: M = 5.1 years, SD = 6.1, non-SC-using: M = 7.4 years, SD = 5.5, *t*(196) = 2.9, *p* < 0.01], showed lower rates (41.9 vs. 59.7%) of methadone/buprenorphine-naloxone street-buying [χ2 (1, *N* = 177) = 4.2, *p* < 0.05] and lower rates (37.2 vs. 61.9%) of methadone/buprenorphine-naloxone injecting [χ2 (1, *N* = 177) = 8.1, *p* < 0.01]. Lower rates (22.7%) of treatment retention was observed among SC using OST clients [χ2 (1, *N* = 198) = 5.2, *p* < 0.05] as compared to those without a history of past year SC consumption (41.6%) at 4-years follow-up. Clients receiving either methadone or buprenorphine-naloxone were similarly compared in terms of their age, gender, years spent in treatment, highest academic degree, SES, occupation, taking other medication (e.g., benzodiazepine), rate of successful dose reduction during treatment, IV use of OST medication, SC use during treatment. Those receiving buprenorphine-naloxone have spent less years in treatment [methadone clients: M = 7.2, SD = 5.7, buprenorphine-naloxone clients: M = 3.6, SD = 4.5, *t*(198) = 3.4, *p* < 0.01], but did not differ from methadone clients in any other measures.

### Substance Use Profiles

Past month and past year SC use frequencies are presented in Table 2.

**TABLE 2:** Self-reported past year and past month SC use.

	**Past year**	**Past month**
Mephedrone *N* (% of full sample)	21 (10.7)	3 (1.5)
Pentedrone *N* (% of full sample)	36 (18.2)	10 (5.1)
MDPV *N* (% of full sample)	16 (8.1)	3 (1.5)
SC use in general *N* (% of the full sample)	44 (22)	12 (6.1)

In order to increase the sample size of SC using clients’ subgroup, we decided to merge distinct past year SC use categories (mephedrone, pentedrone, MDPV) into one SC category (*N* = 44) for further analyses. The overall sample size of the merged subgroup was lower than the sum of the three categories as some of the clients used more than one SC derivative. Nevertheless, the most frequently used SC was pentedrone among our OST clients. Intravenous SC use was identified in a high proportion of past year SC users, with altogether 28 participants (63.6%) reported SC injecting.

Last year use of opiate, cocaine, amphetamine, MDMA, cannabis/hashish and the misuse of benzodiazepines during OST was identified by urine test results. THC was found in 92 samples (46.5%), opiates in 86 (43.4%), benzodiazepine in 31 (15.7%), amphetamine in 19 (9.6%), MDMA in 8 (4%), and cocaine in 6 (3%).

**FIGURE 1:**
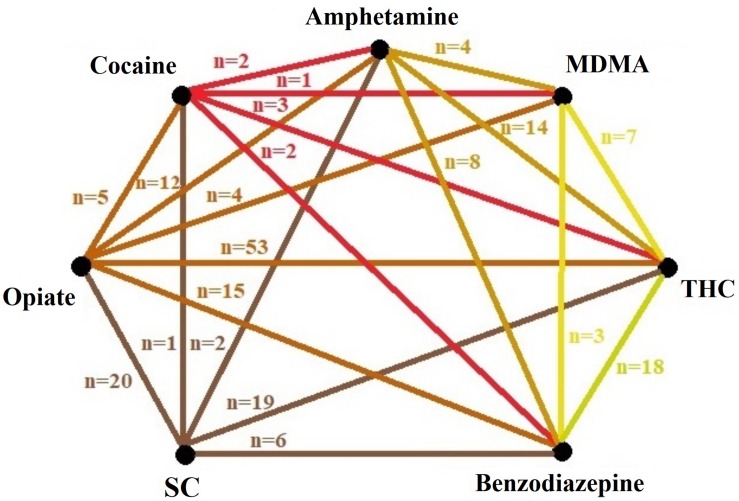
Profile of past year’s concurrent substance use based on urine test results and self-reports (in case of SC). SC, synthetic cannabinoids; MDMA, methylenedioxymethamphetamine; THC, tetrahydrocannabinol.

The current study also aimed to explore past year concurrent substance use patterns during treatment. As self-reports were based on a 1-year recall period, only urine samples were able to provide objective measures regarding co-occurring substance use. But since our study focuses on SC using clients, Figure 1 presents the association between self-reported past year SC use and urine sample-based concomitant substance use profiles as well.

Past year SC use was mainly associated with opiate (*n* = 20) and THC (*n* = 19) consumption in the same year, but not necessarily in the same time period. Regarding concomitant substance use patterns, opiates were most commonly co-ingested with cannabis/hashish (53 clients concurrently used these substances), while among those clients with a history of past year SC use, the consumption of any other stimulants was uncharacteristic. The co-occurrence of more than one stimulant in the urine samples was also rare, indicating that OST clients don’t often mix substances from the same drug class. There were no significant differences in the rate of positive urine samples between SC and non-SC-using clients.

### Childhood Traumatic Experiences

Synthetic cathinone using and non-SC using OST clients were compared regarding their CTQ. Table 3 summarizes the results of comparative statistics.

**TABLE 3:** Childhood traumatic experiences among OST clients with and without a history of past year SC use.

	**SC use**	**SC use not**	**Mann**	
	**reported**	**reported**	**Whitney**	**Effect**
	***N* = 44**	***N* = 154**	***U* test (*U*)**	**size (*r*)**
Emotional neglect Mean (SD)	4.1 (5.9)	5.6 (6.7)	2384.5	0.12
Emotional abuse Mean (SD)	2.7 (4.3)	3.8 (5.2)	2454	0.11
Physical abuse Mean (SD)	2.2 (3.9)	2.6 (4.6)	2832	0.05
Physical neglect Mean (SD)	0.7 (1.3)	2.5 (4.5)	2302.5*	0.26
Sexual abuse Mean (SD)	0.2 (0.7)	0.9 (3.2)	2820.5	0.15

*p < 0.05.

Potential gender differences in the frequency of CTQ were also analyzed, however, there were no significant differences between males and females.

### Cognitive Emotion Regulation Strategies

As for cognitive emotion regulation strategies in the SC and non-SC using subgroups, only one scale (Refocus on planning) showed significant difference between these subgroups (Table 4).

**TABLE 4:** Cognitive emotion regulation related to stressful live events in SC and non-SC using subgroups.

	**SC use**	**SC use not**	**Mann**	
	**reported**	**reported**	**Whitney**	**Effect**
	***N* = 43**	***N* = 133**	***U* test (*U*)**	**size (*r*)**
Self-blame Mean (SD)	5.6 (1.9)	6.3 (2.3)	2381.5	0.17
Other-blame Mean (SD)	3.5 (1.4)	3.5 (1.5)	2747	0.00
Rumination Mean (SD)	5.6 (2)	5.9 (2.4)	2646.5	0.07
Catastrophizing Mean (SD)	4.2 (2.2)	4.5 (2.3)	2582	0.07
Positive refocusing Mean (SD)	4.1 (2.3)	4.3 (2.1)	2632	0.05
Refocus on planning Mean (SD)	5.9 (1.9)	6.9 (2)	2076.5**	0.25
Positive reappraisal Mean (SD)	6.6 (1.8)	7 (2.1)	2549	0.10
Putting into perspective Mean (SD)	5.9 (1.7)	6.1 (1.9)	2766.5	0.06
Acceptance Mean (SD)	5.8 (1.9)	6.1 (2.3)	2618	0.07
Adaptive strategies Mean (SD)	5.7 (1.2)	6.1 (1.4)	2343	0.15
Non-adaptive strategies Mean (SD)	4.7 (1.2)	5.1 (1.4)	2492	0.15

**p < 0.01.

Those clients without a past year history of SC use were more prone to use planning as an adaptive strategy to cope with stressful live situations. Refocus on planning as assessed by the CERQ refers to thinking about potential ways to handle a frustrating event.

**FIGURE 2:**
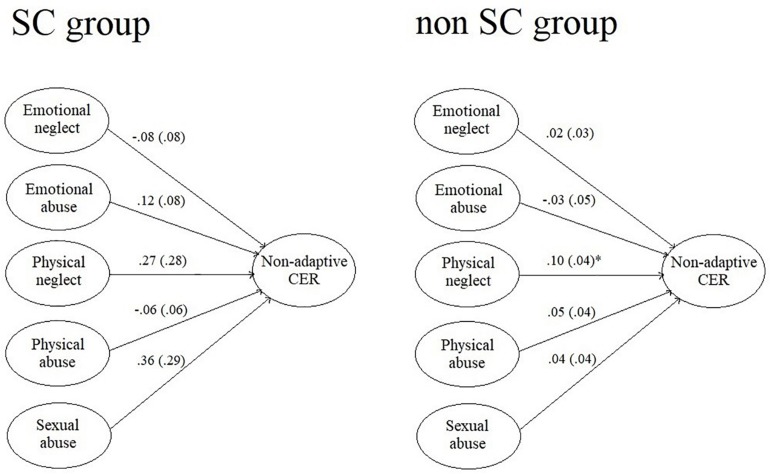
The variability of non-adaptive cognitive emotion regulation strategies explained by childhood traumatic experiences among SC and non-SC-using clients. ^∗^*p* < 0.05; unstandardized *B* coefficients are presented with standard errors (in brackets). SC, synthetic cannabinoids; CER, cognitive emotion regulation.

The association between the type and frequency of endured CTQ and non-adaptive cognitive emotion regulation strategies were analyzed by regression models and compared by past year SC use as a grouping variable. Figure 2 presents the results of the two regression models.

In the non-SC-using subgroup, childhood physical neglect showed significant association with non-adaptive cognitive emotion regulation strategies, although only a low proportion of the outcome variable’s variance was explained by the models (*R*^2^ = 0.15 and 0.19).

### Motivation to Change

In terms of motivation for behavior change, SC-using clients were more likely to show higher scores on the precontemplation domain of the URICA (Table 5).

**TABLE 5:** Stages of motivation for behavior change in SC and non-SC using OST clients.

	**SC use**	**SC use not**	**Mann**	
	**reported**	**reported**	**Whitney**	**Effect**
	***N* = 42**	***N* = 132**	***U* test (*U*)**	**size (*r*)**
Precontemplation Mean (SD)	18.6 (5.9)	16.6 (4.9)	2200.5*	0.18
Contemplation Mean (SD)	30.4 (5.1)	30.7 (7.5)	2845	0.02
Action Mean (SD)	30.1 (6.9)	30.7 (5.9)	2764.5	0.05
Maintenance Mean (SD)	27.6 (6.3)	28.6 (6.9)	2558	0.08
Readiness to change Mean (SD)	17.4 (4.3)	18.3 (4.2)	2416	0.11

*p < 0.05.

This result indicates that SC-using clients might be less motivated to change their substance use habits. This might also be associated with lower treatment retention rates among SC-using participants.

### Psychiatric Symptom Profiles

Psychiatric symptoms were also measured and compared between the two subgroups. Table 6 presents the results.

**TABLE 6:** Psychiatric symptoms of SC and non-SC using subgroups.

	**SC use**	**SC use not**	**Mann**	
	**reported**	**reported**	**Whitney**	**Effect**
	***N* = 43**	***N* = 154**	***U* test(*U*)**	**size (*r*)**
Somatization Mean (SD)	1.3 (1.1)	1.1 (0.9)	2990	0.09
Obsession-Compulsion Mean (SD)	1.4 (0.9)	0.9 (0.8)	2250**	0.28
Interpersonal Sensitivity Mean (SD)	1.4 (1.1)	0.8 (0.8)	2299**	0.29
Depression Mean (SD)	1.8 (1.3)	1.2 (0.9)	2492*	0.26
Anxiety Mean (SD)	1.5 (0.9)	0.9 (0.9)	2357.5**	0.32
Hostility Mean (SD)	1.1 (1.1)	0.8 (0.9)	2594.5	0.15
Phobic Anxiety Mean (SD)	1.2 (1.1)	0.7 (0.8)	2599.5*	0.25
Paranoid Ideation Mean (SD)	1.3 (0.8)	0.9 (0.7)	2271.5**	0.26
Psychoticism Mean (SD)	0.9 (0.9)	0.5 (0.6)	2467***	0.25
Global Severity Index Mean (SD)	1.3 (0.9)	0.9 (0.7)	2220**	0.24

*p < 0.05, **p < 0.01, ***p < 0.001.

**FIGURE 3:**
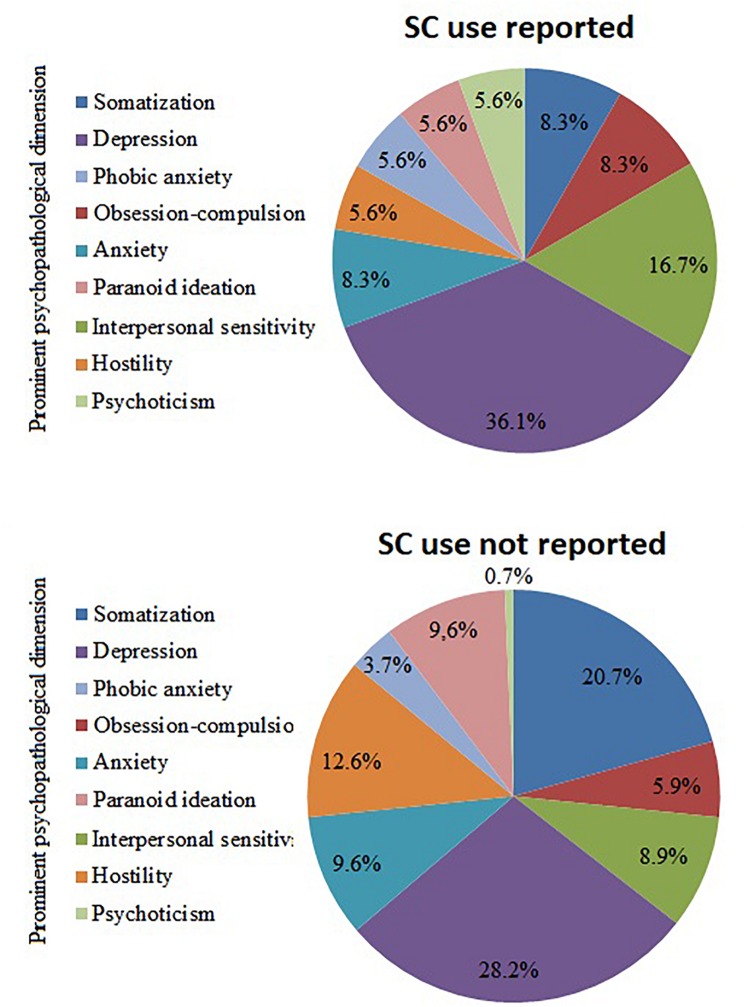
Distribution of prominent psychopathological dimensions with and without reported SC use. SC, synthetic cannabinoids.

Synthetic cathinone-using OST clients showed higher scores on almost every psychiatric symptom scales with the exception of Somatization and Hostility. Furthermore, applying a 63 or above GSI T (raw) score as cut-off for identifying cases of clinical severity, a higher rate of screened psychiatric patients were found among SC (*N* = 22, 53.7%) than non-SC-using (*N* = 39, 25.7%) respondents [χ2 (1, *N* = 193) = 11.7, *p* < 0.01]. As a next step, participants were classified on the basis of their prominent psychopathological dimension, according to the highest standardized *z* score achieved on the BSI factors. The three most dominant dimensions were depression (*n* = 51, 25.8%), somatization (*n* = 15.7%) and hostility (*n* = 19, 9.6%). Figure 3 presents the distribution of the dominant psychopathological dimensions within the subgroups of SC-using and non-SC-using respondents.

When compared, SC- and non-SC-using using subgroups did not differ significantly in the rate of any prominent psychopathological dimensions: Somatization [χ2 (1, *N* = 171) = 2.9, *p* > 0.05], Obsession-compulsion [χ2 (1, *N* = 171) = 0.3, *p* > 0.05], Interpersonal sensitivity [χ2 (1, *N* = 171) = 1.8, *p* > 0.05], Depression [χ2 (1, *N* = 171) = 0.9, *p* > 0.05], Anxiety [χ2 (1, *N* = 171) = 0.1, *p* > 0.05], Hostility [χ2 (1, *N* = 171) = 1.4, *p* > 0.05], Phobic anxiety [χ2 (1, *N* = 171) = 0.2, *p* > 0.05], Paranoid ideation [χ2 (1, *N* = 171) = 0.6, *p* > 0.05], Psychoticism [χ2 (1, *N* = 171) = 3.8, *p* > 0.05]. Furthermore, none of the dominant psychopathological dimensions showed significant association with treatment retention rates, indicating that psychiatric subtypes among OST clients might not differ in their treatment outcomes.

**FIGURE 4:**
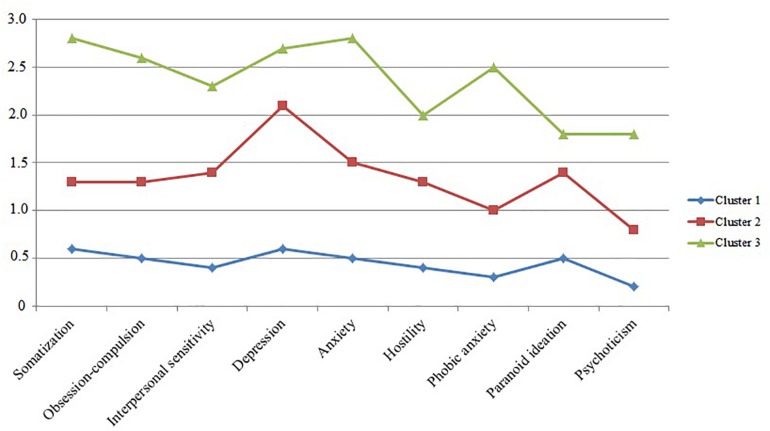
Latent profiles of psychiatric symptom severities.

A person-centered approach was also applied in order to examine potential latent classes of users regarding their psychopathological profile based on achieved BSI factor scores. Latent profile analysis is a form of latent variable analyses (e.g., Collins and Lanza, 2010) with categorical latent variable and continuous manifest indicators (in this case the BSI factor scores). The Bayesian information criteria parsimony index (BIC), entropy and the interpretability of clusters were used during the process of determining the number of latent classes. Lower BIC value and higher value of entropy are preferable in model selection. The determination of the number of classes is usually supported by the results of the likelihood-ratio difference test [Lo–Mendell–Rubin adjusted likelihood-ratio test (LRT)], which compares the estimated model with a model of one less class (*k -* 1). In this case low *p* value (*p* < 0.05) indicates that the model with one less class is rejected in favor of the estimated model, however, none of our tested models yielded significant LRT test, therefore final model determination was based on BIC and entropy values. One- (BIC = 121420.8), two- (BIC = 123256.6), three- (121481.3), and four-class (BIC = 121534.1) solutions were estimated throughout the analysis. Entropy increased at two-class (0.887), reached its peak at three-class (0.947) and decreased at four-class solution (0.483). Finally, a three-class solution was accepted. Figure 4 presents the psychiatric symptom profiles of the three classes.

Based on their most likely latent class membership, 111 participants belonged to the first class (less severe symptomatology), 57 participants to the second class (moderate symptom severity), while 25 respondents belonged to the third class (most severe symptomatology). These symptom profiles differ in their global severity but not in the pattern of dominant psychopathologies, indicating that OST clients might be similar in terms of psychiatric symptom patterns. SC using clients were more likely to belong to either the second (*n* = 16, 39%) or the third class (*n* = 8, 19.5%) than non-SC-using participants (second class: *n* = 41, 27%, third class: *n* = 17, 11.2%) [χ2 (1, *N* = 193) = 5.5, *p* < 0.05]. Clients receiving methadone vs. buprenorphine-naloxone did not differ in the rate of their class memberships [χ2 (1, *N* = 193) = 1.4, *p* > 0.05]. Additionally, there were no differences in treatment retention rates between the members of the three latent classes (the retention rates for the first, second and third class were 38.7, 42.1, and 24%, respectively) [χ2 (2, *N* = 193) = 2.5, *p* > 0.05].

Finally, class membership was predicted by using logistic regression analysis, entering treatment indices and SC use during treatment as covariates in the model. Table 7 summarizes the result of these analyses. The only significant predictor was SC use during treatment regarding first latent class membership, indicating that treatment indices (such as OST medication type, dose, years in treatment or receiving other medication such as benzodiazepines) do not show significant association with the severity of psychiatric symptom profiles. Those clients, however, who used SC during treatment had lower odds of belonging to the less severe psychiatric symptom class.

**TABLE 7:** Treatment indices and SC use during treatment as predictors of latent class memberships.

			**Odds**	
	***B* (SE)**	***p***	**ratio**	**95% CI**

**Class 1 membership**	***R*^2^ = 0.052 (Cox and Snell),**			
	**0.070 (Nagelkerke), *N* = 173**			
OST medication dose	−0.01-(0.01)	0.671	0.99	0.99, 1.01
Years in treatment	−0.04-(0.03)	0.154	0.96	0.90, 1.02
OST medication: methadone vs. buprenorphine-naloxone	−0.97-(0.64)	0.129	0.38	0.11, 1.33
Receiving other medication (e.g., benzodiazepines)	−0.23-(0.32)	0.478	0.79	0.42, 1.51
**SC use during treatment**	−0.94-(0.39)	0.018	**0.39**	0.18, 0.85

**Class 2 membership**	***R*^2^ = 0.036 (Cox and Snell),**			
	**0.051 (Nagelkerke), *N* = 173**			

OST medication dose	−0.00-(0.01)	0.916	0.99	0.99, 1.01
Years in treatment	0.02-(0.03)	0.472	1.02	0.96, 1.09
OST medication: methadone vs. buprenorphine-naloxone	0.84-(0.65)	0.196	2.32	0.65, 8.29
Receiving other medication (e.g., benzodiazepines)	0.43-(0.35)	0.220	1.54	0.77, 3.08
SC use during treatment	0.67-(0.41)	0.100	1.94	0.88, 4.29

**Class 3 membership**	***R*^2^ = 0.023 (Cox and Snell),**			
	**0.045 (Nagelkerke), *N* = 173**			

OST medication dose	0.01-(0.01)	0.470	1.01	0.99, 1.02
Years in treatment	0.05-(0.05)	0.269	1.05	0.96, 1.15
OST medication: methadone vs. buprenorphine-naloxone	0.27-(0.99)	0.783	1.31	0.19, 9.05
Receiving other medication (e.g., benzodiazepines)	−0.32-(0.48)	0.513	0.73	0.28, 1.88
SC use during treatment	0.71-(0.54)	0.189	2.04	0.70, 5.91

*Significant explanatory variables and related values are boldfaced. CI, confidence interval.*

### Predictors of SC Use

In order to examine the association between past year SC use and those variables that resulted in significant differences between SC-using and non-SC-using subgroups, a logistic regression model was tested (Table 8).

**TABLE 8:** Logistic regression model to test the retrospective odds for past year SC use.

			**Odds**	
	***B* (SE)**	***p***	**ratio**	**95% CI**
***R*^2^ = 0.179 (Cox and Snell), 0.271 (Nagelkerke), *N* = 168**				
Age	−0.05-(0.03)	0.142	0.95	0.89, 1.02
**Years in treatment**	−0.09-(0.04)	0.034	**0.91**	0.84, 0.99
Childhood physical neglect (CTQ)	−0.15-(0.09)	0.090	0.86	0.72, 1.03
Refocus on planning (CERQ)	−0.16-(0.10)	0.104	0.85	0.69, 1.03
Precontemplation (URICA)	0.06-(0.04)	0.133	1.06	0.98, 1.14
**Global Severity Index (BSI)**	0.71-(0.29)	0.014	**2.03**	1.16, 3.56

*Significant explanatory variables and related values are boldfaced. CI, confidence interval.*

Clients with more severe psychiatric symptoms showed elevated risk for past year SC use (OR = 2.03), while those who spent longer time in treatment had lower odds of SC consumption (OR = 0.91).

### Predictors of Treatment Retention

Finally, a logistic regression model was applied to test potential explanatory variables of treatment retention. Besides SC use, explanatory variables (age, gender, years in treatment, OST medication dose) were selected as commonly reported factors associated with treatment retention. Table 9 summarizes the results.

**TABLE 9:** A logistic regression model to predict treatment retention.

			**Odds**	
	***B* (SE)**	***p***	**ratio**	**95% CI**
***R*^2^ = 0.056 (Cox and Snell), 0.057 (Nagelkerke), *N* = 198**				
Age	−0.02-(0.03)	0.465	0.98	0.94, 1.03
Gender	−0.25-(0.35)	0.477	0.78	0.39, 1.54
Years in treatment	−0.05-(0.03)	0.093	1.05	0.99, 1.11
OST medication dose	−0.01-(0.01)	0.253	1.01	0.99, 1.01
**SC use during treatment**	−0.82-(0.42)	0.049	**0.44**	0.19, 0.99

*Significant explanatory variables and related values are boldfaced. CI, confidence interval.*

Past year SC use was the only significant predictor in the model, associated with decreased treatment retention odds (OR = 0.44).

## Discussion

Our study indicated differences between OST clients with and without a history of past year SC use in terms of treatment indices, childhood trauma experiences, cognitive emotion regulation strategies, motivation for behavior change and psychiatric symptom severity.

As regards treatment indices, the result that SC-using clients were less likely to buy street methadone/buprenorphine-naloxone as well as to inject their OST medication can primarily be explained by the assumption that instead of misusing/overusing opioid medication they are more prone to use SC in order to experience euphoria during treatment. Clients receiving buprenorphine-naloxone have spent less years in treatment than those who were taking methadone. This finding might be attributed to the fact that Suboxone was only introduced in 2007 in Hungary (Demetrovics et al., 2009), and further years have passed until clinicians started to prescribe this medication instead of methadone as a first-line OST medication. The buprenorphine-naloxone clients participating in this study were most likely among the first new clients who started their OST treatment with buprenorphine-naloxone and not methadone. Within the scope of the current study clients receiving methadone or buprenorphine-naloxone did not differ in any further measures. 4-year retention rate among non-SC-using clients (41.6%) was relatively high as compared to previous findings that identified a 18 months retention rate of 32.3% (Lin et al., 2013), a 1-year retention rate of 34.4% (Sheikh Fathollahi et al., 2016) or a 3-year retention rate of 20% (Yang et al., 2013) among OST clients, although some reported much higher, 87% 1-year retention rate (Jiang et al., 2014) or a 57% retention rate at 4-years follow-up (Wei et al., 2013). Past year SC use was associated with higher proportion of treatment drop-outs. Given the finding that almost every second client was screened positive for past year cannabis or opiate use during treatment and considering that there were no significant differences in the rate of positive urine test results between SC and non-SC-using clients, it might be assumed that SC use *per se* is a significant risk factor for drop-out. This assumption was further supported by the results of the second logistic regression model, namely that past year’s SC use was a significant predictor of reduced treatment retention. SC using OST clients had approximately two times higher risk of drop out. In contrast to previous findings, age, gender, years spent in treatment or the dose of methadone or buprenorphine-naloxone did not show significant association with treatment retention. SC users were more likely to be in the precontemplation stage of behavior change, showing limited or no motivation to stop using psychoactive substances. Therefore frequent screening of clients’ current motivation for behavior change should be an important routine in addiction care and especially in low-threshold services such as OST programs, accompanied by motivational interviewing.

Childhood physical neglect was related to non-adaptive cognitive emotion regulation strategies among non-SC-using clients. Previous results indicate that childhood neglect may predict deficits in the recognition of positive emotions (Young and Widom, 2014), that might explain difficulties in applying adaptive strategies, such as refocus on planning, positive reappraisal or putting a stressful life event into a more acceptable perspective. On a neurobiological level, others found that right amygdale volume mediates the association between neglect and anxiety (Roth et al., 2018) or that early exposure to either emotional or physical neglect is related to amygdala hypertrophy and mood disorders (Herzog and Schmahl, 2018). Increased risk for anxiety and mood disorders thus further reduce the capability to focus on positive outcomes in stressful live situations. Non-SC-using clients reported a more frequent exposure to physical neglect in their childhood, denoting that SC use is probably not a maladaptive self-medication attempt in terms of dealing with early trauma experiences.

Approximately half of SC-using clients were characterized by psychiatric symptoms of clinical severity, and global severity of these symptoms was a significant explanatory variable of SC use during treatment. Considering the identified latent profiles of psychiatric symptom severities, none of the treatment indices could predict class memberships, however, SC use during treatment was significantly associated with lower odds to belong to the less severe class of psychiatric symptoms. Prominent psychopathological dimensions were additionally examined. Our result that the most dominant psychopathologies of opioid-dependent clients are depression, somatization and hostility is in line with former findings, including the self-medication hypothesis of Khantzian (1985) that highlights the powerful muting action of opiates on the threatening affect of aggression. OST clients might also use or misuse their medication as a form of maladaptive self-medication in order to ease their depressive symptoms and violent urges. These findings emphasize the importance of psychiatric screening among OST clients in order to identify patients with potential unrecognized dual diagnoses. On the other hand, unlike Maremmani et al. (2016) we could not identify any significant associations between treatment outcomes and prominent psychopathological subtypes. Nevertheless, as other authors have already pointed out (e.g., Schulte et al., 2010), unidentified comorbid disorders may increase the risk of drop-out from addiction treatment, yet psychiatric comorbidity often remains a latent factor. Another related anomaly also needs to be addressed: according to the results of McGovern et al. (2014), only a minority of addiction treatment programs is capable of providing integrated services. Hence, public access to integrated care should be increased. A possible solution for enhancing the efficacy of dual diagnosis recognition would be the synthesis of addiction/psychiatry and primary care services, stressing the role of GPs and community services in early identification of both addiction problems (among other things, anamnestic information about SC or NPS use in general) and co-occurring mental disorders, including PTSD as well.

### Limitations

The current study is not without limitations. While past year’s opiate, cocaine, amphetamine, MDMA, THC or benzodiazepine misuse were described by positive urine test results as objective measures, SC use was based on clients’ self-reports. Furthermore, past year’s SC use as a retrospective and self-reported measure was predicted by variables that were subsequently assessed (e.g., GSI). In these cases, a relative persistence was assumed regarding the values of explanatory variables, however, this is not necessarily realistic as the severity of psychiatric symptoms, the motivation for behavior change or the current cognitive emotion regulation strategies may markedly change over a time-span of 1 year. As regards our statistical analyses, only small – or in some cases medium – effect sizes were found, as based on Cohen’s rule of thumb. Childhood traumatic experiences were also assessed by clients’ retrospective self-reports, therefore these results might have been impacted by potential recall biases. Finally, OST clients’ psychiatric profiles assessed by BSI could not be supported/confirmed by their clinical diagnoses as all clients of the drug outpatient center received the exact same comorbid diagnoses of opioid dependence (F11.20) and mixed anxiety and depressive disorder (F41.2), most likely due to a rather simplifying diagnostic procedure that mainly justifies the applied pharmacotherapy but does not necessarily aim to provide an elaborate psychopathological profile of the clients.

### Future Directions

This study did not assess PTSD as a potential outcome of childhood trauma, nor the mediatory role of psychiatric symptom severity between trauma exposure and non-adaptive cognitive emotion regulation strategies. These associations were partly tested by others (e.g., Garnefski et al., 2017; McLaughlin and Lambert, 2017; Karatzias et al., 2018), but not in a sample of OST clients or in light of concurrent SC consumption. Differences in emotion regulation and psychopathology between SC-using clients and those who consume other stimulant drugs (e.g., cocaine, amphetamine, MDMA) would highlight further specificities of SC regarding the consecutive neuropsychological traits of its use.

## Conclusion

Synthetic cathinone use is a risk factor of poorer treatment outcomes, characterized by more severe psychiatric symptoms as well as a lack of motivation to change substance use behavior among opioid dependent clients. The availability of rapid urine tests able to detect NPS like SC need to be increased in order to more efficiently screen OST clients’ SC consumption during therapy.

## Data Availability Statement

The datasets generated for this study are available on request to the corresponding author.

## Ethics Statement

The studies involving human participants were reviewed and approved by the Research Ethics Committee of the Nyírõ Gyula National Institute of Psychiatry and Addictions, Budapest, Hungary. The patients/participants provided their written informed consent to participate in this study.

## Author Contributions

MK-F, AK, KK, and JF conceived the presented idea and developed the questionnaire for the study. PP and JH were involved in data collection procedure. All authors discussed the results and contributed to the manuscript. ZD helped in supervising the project. MK-F, AK, KK, JF, and ZD took part in writing the manuscript. MK-F was responsible for statistical analyses.

## Conflict of Interest

The authors declare that the research was conducted in the absence of any commercial or financial relationships that could be construed as a potential conflict of interest.

The handling Editor declared a past co-authorship with one of the authors ZD.

## Abbreviations

4-MEC: 4-methylethcathinone
BSI: Brief Symptom Inventory
CERQ: Cognitive Emotion Regulation Questionnaire
CTQ-SF: Childhood Trauma Questionnaire
GSI: Global Severity Index
MDPV: 3,4-methylenedioxypyrovalerone
mephedrone: 4-methylmethcathinone
methylone: 3,4-methylenedioxy-*N*-methylcathinone
NPS: novel psychoactive substance(s)
OST: opioid substitution treatment
pentedrone: 2-(methylamino)-1-phenylpentan-1-one
PTG: posttraumatic growth
PTSD: posttraumatic stress disorder
SC: synthetic cathinone(s)
SES: socioeconomic status
SUD: substance use disorder(s)
TTM: Transtheoretical Model
URICA: University of Rhode Island Change Assessment Scale.
